# Supplementary material for: Meta-Analysis and Gene Set Enrichment Relative to ER Status Reveal Elevated Activity of MYC and E2F in the “Basal” Breast Cancer Subgroup
Source: PLoS One. 2009 Mar 9;4(3):e4710. doi: 10.1371/journal.pone.0004710 (PMC2650420; doi:10.1371/journal.pone.0004710)
Supplement: Dataset S1 — GSEA results from first screen. The file contains a zipped archive of the HTML results of the second GSEA screen. Reports for enriched or depleted datasets may be accessed from the “index.html” link within the my_analysis.GseaPreranked.1219975950625.rpt.zip ZIP archive. Enrichment plots are available for each of the 50 most enriched and depleted gene sets. (13.32 MB ZIP) [file pone.0004710.s001.zip › my_analysis.GseaPreranked.1219975950625.rpt.zip!//SHEPARD_GENES_COMMON_BW_CB_MO.html]

Details for gene set SHEPARD\_GENES\_COMMON\_BW\_CB\_MO[GSEA]

|  || Dataset | zSco.ERneg.to.ERpos\_collapsed |
| Phenotype | NoPhenotypeAvailable |
| Upregulated in class | na\_pos |
| GeneSet | SHEPARD\_GENES\_COMMON\_BW\_CB\_MO |
| Enrichment Score (ES) | 0.6111463 |
| Normalized Enrichment Score (NES) | 2.3043454 |
| Nominal p-value | 0.0 |
| FDR q-value | 0.0 |
| FWER p-Value | 0.0 |
Table: GSEA Results Summary

  

Fig 1: Enrichment plot: SHEPARD\_GENES\_COMMON\_BW\_CB\_MO      
 Profile of the Running ES Score & Positions of GeneSet Members on the Rank Ordered List

  

| PROBE | GENE SYMBOL | GENE\_TITLE | RANK IN GENE LIST | RANK METRIC SCORE | RUNNING ES | CORE ENRICHMENT || 1 | CDC20 |  |  | 9 | 7.151 | 0.0389 | Yes |
| 2 | TTK |  |  | 11 | 7.059 | 0.0780 | Yes |
| 3 | FOXM1 |  |  | 21 | 6.760 | 0.1148 | Yes |
| 4 | SFRP1 |  |  | 44 | 6.369 | 0.1484 | Yes |
| 5 | AURKB |  |  | 76 | 6.038 | 0.1795 | Yes |
| 6 | MKI67 |  |  | 98 | 5.871 | 0.2105 | Yes |
| 7 | GTSE1 |  |  | 129 | 5.694 | 0.2398 | Yes |
| 8 | MELK |  |  | 181 | 5.424 | 0.2660 | Yes |
| 9 | KIF14 |  |  | 221 | 5.215 | 0.2919 | Yes |
| 10 | BIRC5 |  |  | 229 | 5.191 | 0.3202 | Yes |
| 11 | TPX2 |  |  | 231 | 5.180 | 0.3488 | Yes |
| 12 | HMGA1 |  |  | 244 | 5.125 | 0.3763 | Yes |
| 13 | SOX11 |  |  | 250 | 5.094 | 0.4041 | Yes |
| 14 | CDC14B |  |  | 280 | 4.994 | 0.4296 | Yes |
| 15 | CDCA3 |  |  | 321 | 4.868 | 0.4536 | Yes |
| 16 | ASPM |  |  | 359 | 4.767 | 0.4772 | Yes |
| 17 | DLG7 |  |  | 521 | 4.306 | 0.4889 | Yes |
| 18 | PRC1 |  |  | 543 | 4.253 | 0.5109 | Yes |
| 19 | TACC3 |  |  | 669 | 3.995 | 0.5236 | Yes |
| 20 | KIF11 |  |  | 713 | 3.909 | 0.5421 | Yes |
| 21 | BMS1L |  |  | 1028 | 3.446 | 0.5375 | Yes |
| 22 | KIF23 |  |  | 1030 | 3.442 | 0.5565 | Yes |
| 23 | CDC2 |  |  | 1259 | 3.182 | 0.5569 | Yes |
| 24 | FBXO5 |  |  | 1304 | 3.137 | 0.5710 | Yes |
| 25 | ATP1A1 |  |  | 1390 | 3.048 | 0.5815 | Yes |
| 26 | CKAP2 |  |  | 1492 | 2.951 | 0.5902 | Yes |
| 27 | CCNB1 |  |  | 1626 | 2.823 | 0.5958 | Yes |
| 28 | NOVA2 |  |  | 1786 | 2.704 | 0.5988 | Yes |
| 29 | ELAVL4 |  |  | 1820 | 2.678 | 0.6111 | Yes |
| 30 | HLX1 |  |  | 2213 | 2.380 | 0.5948 | No |
| 31 | TK1 |  |  | 2730 | 2.076 | 0.5674 | No |
| 32 | PLP1 |  |  | 2842 | 2.019 | 0.5702 | No |
| 33 | TNXB |  |  | 3236 | 1.822 | 0.5506 | No |
| 34 | CTSL |  |  | 3453 | 1.729 | 0.5439 | No |
| 35 | HEY1 |  |  | 3698 | 1.621 | 0.5345 | No |
| 36 | GRIK3 |  |  | 3864 | 1.567 | 0.5308 | No |
| 37 | TTN |  |  | 4935 | 1.176 | 0.4566 | No |
| 38 | MYH8 |  |  | 4956 | 1.170 | 0.4616 | No |
| 39 | MT2A |  |  | 5023 | 1.144 | 0.4629 | No |
| 40 | NOTCH2 |  |  | 5101 | 1.116 | 0.4633 | No |
| 41 | FLJ12547 |  |  | 5436 | 0.999 | 0.4436 | No |
| 42 | POU3F1 |  |  | 5694 | 0.914 | 0.4293 | No |
| 43 | POU3F2 |  |  | 5743 | 0.898 | 0.4307 | No |
| 44 | POU3F3 |  |  | 5942 | 0.834 | 0.4204 | No |
| 45 | SOX21 |  |  | 6441 | 0.670 | 0.3865 | No |
| 46 | ASCL1 |  |  | 6905 | 0.517 | 0.3545 | No |
| 47 | MDK |  |  | 8042 | 0.089 | 0.2693 | No |
| 48 | LHX1 |  |  | 8257 | 0.013 | 0.2532 | No |
| 49 | PTPRN |  |  | 8730 | -0.181 | 0.2187 | No |
| 50 | EDG1 |  |  | 9268 | -0.440 | 0.1806 | No |
| 51 | FGFR4 |  |  | 9832 | -0.745 | 0.1423 | No |
| 52 | DDX17 |  |  | 9961 | -0.821 | 0.1372 | No |
| 53 | ARID5B |  |  | 10415 | -1.097 | 0.1091 | No |
| 54 | NR2F1 |  |  | 10730 | -1.335 | 0.0928 | No |
| 55 | NUMA1 |  |  | 12034 | -2.614 | 0.0090 | No |
| 56 | UCP2 |  |  | 12270 | -2.930 | 0.0075 | No |
| 57 | TNRC9 |  |  | 12280 | -2.941 | 0.0232 | No |
| 58 | GOLGB1 |  |  | 12478 | -3.228 | 0.0262 | No |
| 59 | TM7SF2 |  |  | 12522 | -3.295 | 0.0412 | No |
| 60 | NDE1 |  |  | 12586 | -3.408 | 0.0554 | No |
Table: GSEA details [plain text format]

  

Fig 2: SHEPARD\_GENES\_COMMON\_BW\_CB\_MO: Random ES distribution      
 Gene set null distribution of ES for **SHEPARD\_GENES\_COMMON\_BW\_CB\_MO**
